# Dr. Haslam on the Intellectual Composition of Man

**Published:** 1828-04-01

**Authors:** 


*Lectures on the Intellectual Composition of Man. (Delivered at the London Medical Society.)* By Dr. Haslam.

Dr. Haslam, by a long residence as house-apothecary in Bedlam, where men are said to be "out of their minds," must have had extensive opportunities of communing with the "disembodied *spirit*," and studying all the sublime attributes of the immortal mind. Where, indeed, could a philosopher find such excellent materials for his metaphysical researches as—

In those deep solitudes and awful *cells*,
Where heavenly pensive contemplation dwells,
And ever musing *melancholy* reigns?

We have said the *immortal mind*—and happy are we to have the authority of Dr. Haslam for this doctrine of immortality. "If any one, (says he) should suspect that a wall is here built up, to which the branches of infidelity may be trained; and which forms a line of separation from the orthodoxy that is accredited—or if he presumes that a hopeless materialism will be disseminated, he will be wholly disappointed." Again, he says that, as we "penetrate more intimately into the substance, (of the lectures) they will be found to afford additional reasons, and, I think convincing proofs, of the immediate and *future responsibility* of man." Dr. Haslam stands fair to be made a bishop, if he preaches up such doctrines as these! How cheering is this assurance, after the following somewhat different sentiment delivered by Mr. Lawrence.

"If the intellectual phenomena of man require an *immaterial principle* superadded to the brain, we must equally concede it to the more rational animals. If we grant it to these, we cannot refuse it to the next in order, and so on in succession to the whole series—to the oyster, the sea æenemone, the polype, the microscopic animalcules. Is any one prepared to admit the existence of *immaterial* principles in all these cases? If not, he must equally reject it in *man*."—*Physiol. Lect. p.* 110.

When we came to examine Dr. Haslam's doctrines, however, we found nothing that supported these brilliant expectations of future existence.

"The human mind is not the progressive unfolding of *intellectual* germs, which Nature first protrudes and subsequently expands; but a *structure* that is reared, in its primordium, by casual excitations, and in its most important attainments, by the active exertions of the individual himself."

Now what is all this laboured definition but a new version of the theory of Locke, who likened the human mind to a blank sheet of paper, on which the ideas were afterwards written by the senses? Did not Newton ask—"Is not the sensorium of animals, the place where the sentient substance is present, and to which the sensible species of things are brought through the nerves and brain, that there they may be perceived by the mind present in that place?" Is it not the doctrine of Priestly somewhat mystified, who argues that "all our ideas either proceed from the bodily senses, or are consequent upon the perceptions of sense." Finally, is it not a periphrasis of Mr. Lawrence, who tell us, that the mind is built up before our eyes by the senses? There is nothing, in fact, so easy now-a-days, as to appear a great original by bringing forward old matter with a new face. Most physiologists, we apprehend, were acquainted with the fact that, in addition to those organs which carry on the circulation, respiration, digestion, secretion, and other processes, there was an apparatus called a brain and nervous system, by which man became acquainted, and held constant converse with the world around him. But we were mistaken. This grand discovery was to issue from a narrow, dark, and somewhat odoriferous court in Fleet-street, in the year 1827.

"Man is not a mere corporeal and animated mass, not exclusively the compound of circulation, respiration, digestion, secretion, and other vital actions: this living system is endowed with organs and capacities of a different and more elevated nature; susceptibilities of impression, and capacities of intelligence, that, by proper culture and application, may become gradually ripened into the display of mind."

Dr. H. then goes on to inform his audience and the world, that, in a state of "perfect formation," we are furnished with five senses—that we have eyes, ears, a nose, a tongue, and "pulpy *internal extremities* of the fingers," which compose the organ of touch. We are really at a loss to conceive what is meant by "the *internal extremity*" of a finger! We quite agree with Dr. Haslam, however, that no proof has ever been produced "that the infant brought with it any *memorial* of its *uterine* existence." We should think, indeed, that "the pulpy *internal extremities*" of its fingers were but badly calculated for making memoranda, during its uterine existence. We are next informed that light impresses the eye, sound the ear, resistance impresses the touch, &c. We, also, subscribe to the observation, that, "it is impossible to describe the *imperceptible* gradations of sight," during the first weeks of our extra-uterine existence. The Doctor's observations on perception contain nothing but what is known to the merest tyro in physiology, though they are delivered with an air of oracular originality that must excite a smile. There are some observations, under the head of perception, on which we shall take the liberty to make a comment or two. Dr. H. says, the organs of sense are fatigued by exertion, in the same way as the voluntary muscles, and recruited by rest, &c. This renovation appears to the orator a "perfect mystery"—not more perfect, we should imagine, than secretion, digestion, or any other process in the animal economy. "There are, however, *slight glimmerings* that *seem* to afford some clue to *conjecture*." Here our hopes were kindled, but an extinguisher was soon placed over them. The *voluntary* muscles are liable to fatigue and require alternations of *repose*; but the involuntary muscles are never tired, and "the heart will continue its pulsations for a century without any *individual* feeling of weariness." Now let us see how this learned philosopher is supported by facts. Upon the most accurate calculation that can be made, the *contraction* of the ventricles of the heart occupies *one third* of a second, and the *relaxation* the other *two thirds*, when the circulation is at 60 beats in the minute. The same ratio prevails, whether the heart's action be retarded or accelerated. Thus, then, the muscular fibres of the heart *act* eight hours out of the 24 hours—and are *passive* during the space of 16 hours in the same period. Yet this sapient philosopher represents the heart, as an *involuntary* muscle, as capable of continuing its action *uninterruptedly* for a century, without any *individual* feeling of weariness! If we examine the action of all other involuntary muscles, as those of the stomach, intestines, &c., we find the same alternations of labour and rest—and that Nature has not imposed on them any severer task, or endued them with any power of *perpetual motion* beyond other muscles in the human frame. Such are the *slight glimmerings* affording clues to *conjecture*, in the dark passages of Bolt Court!

*Memory.*

Dr. Haslam has occupied a prodigious quantity of verbiage on this mysterious power, without letting in a single *glimmering* that can afford a clue to *conjecture* as to its nature. We apprehend that two or three words might comprise the whole of what we know of memory. It is a record of perceptions, and reflexions, with the power of calling up these records to the mind's eye, voluntarily or involuntarily. We cannot entirely agree with our learned metaphysician in the following passage. "We are fully aware of the directing wisdom that constituted memory the associate of perception, *because perception alone would have conveyed no intelligence*." Now we are inclined to think, that *perception* does convey *intelligence*, whether that intelligence be recorded by memory or not. Suppose Dr. Haslam was transported (we merely mean an imaginary flight) to Botany Bay, and then, for the first time, to behold a KANGAROO hopping about. Would his *perceptions* of this animal convey no *intelligence*, as to figure, colour, shape, &c., even if his memory were extremely defective? The doctrine, we think, is not quite sound. We see men in very advanced age, whose memories are almost completely annihilated, and who yet have clear perceptions of those objects that are presented to their senses at the moment, and reason on them accurately. The next moment they recollect nothing of what has passed. Memory greatly enhances, no doubt, the value of perceptions, but perceptions, we imagine, convey intelligence, whether they are treasured in the memory or not.

In the perusal of these lectures, we have not been able to collect any more distinct views of the intellectual composition of man than we before possessed —and we are quite sure that, in many instances, Dr. Haslam's attempts to unravel the mysteries of mind, have only tended to make obscurium obscurius. "I should define *thought*" says he "to be that operation of the intellect, whereby it elaborates its peculiar and intrinsic attainments." If any man can form a clearer idea of thought by this definition, he must have a penetrating mind indeed. It is neither more nor less than—thought is thought!

"It has been considered altogether a *spiritual* process, and it has likewise been held, that any other manner of interpretation, would introduce and countenance materialism. This is a most mistaken and absurd inference, and appears to have arisen from our ignorance of the actual nature of the process of thought. In a former lecture it has been explained, that all the living functions, and all the intellectual capacities on which the mind is built up, are to be considered as the endowments of a divine cause, and are not to be expounded by any material solution. Ordinary observation will convince us, that the human mind is reared, or formed, by the excitations of the external world, and from its own intrinsic achievements; and the assumption of a spiritual interference would destroy the whole fabric of intellectual physiology, and abolish the responsibility of man."

Thus, living functions are divine endowments, but THOUGHT, the highest function of an ORGANIZED BEING, must be explained without "spiritual interference." In short, dear Doctor, you know as much about the nature of THOUGHT, as you do about the inhabitants of the Moon—nor do *we* profess to be a whit wiser than yourself. You have contrived to clothe a small quantity of old matter in a large number of new words, which are by no means badly strung together. But as to the addition which you have made to our previous stock of knowledge—it is neither here nor there.

